# In Vitro and Ultrastructural Evaluation of the Cytotoxic and Antileishmanial Activities of Thiosemicarbazone Compounds Against Promastigotes and Axenic Amastigotes of *Leishmania infantum*

**DOI:** 10.3390/tropicalmed10110325

**Published:** 2025-11-19

**Authors:** Janderson Weydson Lopes Menezes da Silva, Andréa Regina Alves da Rocha Diniz, Alberon Ribeiro de Araújo, Gabriel Gazzoni Araújo Gonçalves, Dyana Leal Veras, Marton Kaique de Andrade Cavalcante, Jana Messias Sandes, Iranildo José da Cruz Filho, Diego Santa Clara Marques, Maria do Carmo Alves de Lima, Ana Paula Sampaio Feitosa, Luiz Carlos Alves, Fábio André Brayner

**Affiliations:** 1 Laboratório de Doenças Parasitárias, Infecciosas e Não-Infecciosas, Departamento de Parasitologia, Instituto Aggeu Magalhães—IAM-FIOCRUZ, Recife 50740-465, PE, Brazil; jandersonweydson@gmail.com (J.W.L.M.d.S.); andrearegina3005@hotmail.com (A.R.A.d.R.D.); alberon.araujo@fiocruz.br (A.R.d.A.); dyana_leal@yahoo.com.br (D.L.V.); martonc@fiocruz.br (M.K.d.A.C.); luizcarlos.alves@fiocruz.br (L.C.A.); 2 Laboratório de Microscopia Eletrônica, Instituto Keizo Asami—iLIKA-UFPE, Recife 50670-901, PE, Brazil; gabrielgazzoni@gmail.com (G.G.A.G.); jana.sandes@ufpe.br (J.M.S.); 3 Laboratório de Planejamento em Química Medicinal, Departamento de Antibióticos—UFPE, Recife 50670-901, PE, Brazil; iranildoj@gmail.com (I.J.d.C.F.); diego.scmarques@ufpe.br (D.S.C.M.); nenalima.mariadocarmo@gmail.com (M.d.C.A.d.L.); 4 Secretaria de Educação do Rio Grande do Norte, Mossoró 59064-901, RN, Brazil; sampaiofeitosa@hotmail.com; 5 Universidade de Pernambuco—UPE, Campus Mata Norte, Nazaré da Mata 55800-000, PE, Brazil

**Keywords:** thiosemicarbazones, parasite ultrastructure, cytotoxicity, apoptosis, immunomodulation, mitochondrial dysfunction

## Abstract

Leishmaniasis remains a global health challenge, and the search for effective and selective therapeutic agents is crucial. This study evaluated the in vitro antileishmanial and cytotoxic activities of thiosemicarbazone compounds (LT-70, LT-73, LT-75, and LT-89) against *Leishmania infantum* promastigote and axenic amastigote forms. The compounds demonstrated strong leishmanicidal activity, with IC_50_ values ranging from 10.5 to 14 µM. At the lowest tested concentration (20 µM) the compounds produced percent inhibitions of 100% (LT-70), 100% (LT-73), 100% (LT-75) and 100% (LT-89). Cytotoxicity assays on J774.A1 macrophages revealed CC_50_ values from 60 µM to >75 µM, with LT-73 and LT-75 showing low toxicity (CC_50_ > 75µM). Selectivity index (SI) ranged from 7.1 for LT-75 and 5.8 for LT-73, indicating potential for further development. Ultrastructural analysis by SEM and TEM revealed cellular and organelle damage, including membrane rupture and mitochondrial swelling, especially after LT-73 and LT-75 treatment. Immunomodulatory assays indicated induction of TNF-α and IFN-γ production, with significant IL-6 reduction. Flow cytometry data suggest mitochondrial dysfunction and apoptosis-like features, particularly for LT-73. Membrane potential assays suggested mitochondrial depolarization by LT-73. LT thiosemicarbazone derivatives present specific structural modifications that enhance antileishmanial selectivity and reveal a dual mechanism of action combining mitochondrial dysfunction and immunomodulatory effects. These findings support the potential of thiosemicarbazone derivatives as promising antileishmanial agents with selective cytotoxicity and immunomodulatory effects.

## 1. Introduction

Visceral leishmaniasis (VL) is a zoonosis with chronic progression and systemic involvement that can lead to death if not properly treated. It is caused by two protozoan species of the genus *Leishmania*, *Leishmania infantum* and *Leishmania donovani*. These protozoa are responsible for approximately 90,000 reported cases annually, of which 45% are officially registered [1].

Among the factors contributing to the increasing incidence of new VL cases worldwide are: the inefficiency of biological vector control, the lack of strong public policies in endemic regions, the adaptation of the sandfly to urbanized areas, the presence of animals serving as domestic reservoirs, deforestation, various socioeconomic factors, and population migration [2,3].

The treatment of VL requires that each therapeutic alternative be evaluated by considering its chemical properties as well as its benefits and limitations [4]. First-line drugs are the pentavalent antimonials (Sb^5+^), which have rapid action but present systemic side effects such as neurological toxicity, high cardiac and renal toxicity, and lower cost when compared to other drugs used in treatment [5]. Second-line drugs are primarily represented by amphotericin B deoxycholate (AmB-D), which, although a second-choice treatment, has been limited by the spread of resistance and due to chronic and acute adverse effects observed after administration, including fever, chills, nephrotoxicity, hypokalemia, myocarditis, and even death [6]. New formulations have been developed to minimize these effects, which in turn have increased treatment costs [6,7]. Miltefosine is the main third-line drug for VL treatment, which is costly and associated with severe gastrointestinal discomfort [5,8]. Despite the availability of current treatments such as pentavalent antimonials, amphotericin B, and miltefosine, therapeutic management of leishmaniasis remains limited by toxicity, cost, and increasing parasite resistance. Recent research has focused on innovative approaches, including peptide-based agents, metal-complex therapeutics, and multitarget small molecules designed to enhance efficacy and overcome resistance [9,10].

The limited therapeutic options, the significant adverse effects, and the high cost of drugs currently available for VL treatment highlight the need to investigate new alternatives with lower cost and milder side effects [1,2]. Heterocyclic compounds are commercially available for various therapeutic purposes with different pharmacological actions, including the anti-inflammatory and analgesic effects of dipyrone, the antiparasitic activity of metronidazole, and the antiviral action of ribavirin [11,12,13,14].

Several thiosemicarbazone molecules have shown promise in combating *Leishmania* spp. strains resistant to currently available drugs [15,16]. In vivo studies with heterocyclic compounds of classes similar to thiosemicarbazones have also demonstrated good antiparasitic activity against trypanosomatids, with lower cytotoxicity and the ability to significantly reduce parasitic load [17,18,19]. However, despite these advances, such molecules have not yet been approved for clinical use, and their mechanisms of action, toxicity in different biological systems, and efficacy in more robust models still require investigation. These gaps justify the continuation of studies, particularly for the identification of new candidates with improved pharmacological profiles. Previous studies have demonstrated the antileishmanial potential of indol-3-yl-thiosemicarbazone derivatives [16]. However, there remains a need to explore new structural analogs with modified substituents that may influence pharmacological selectivity and biological response.

Despite the availability of drugs such as amphotericin B, pentavalent antimonials and miltefosine, clinical management of visceral leishmaniasis remains constrained by substantial toxicity, prolonged regimens, variable efficacy and the emergence of resistance. Thiosemicarbazones represent a versatile chemical scaffold with multitarget potential and low synthetic cost; however, no thiosemicarbazone derivative has reached clinical approval for leishmaniasis. Therefore, there is an unmet need for rational optimization of these molecules aimed at improving selectivity, reducing toxicity and elucidating mechanisms of action—rationales that motivated the present study on LT derivatives.

## 2. Materials and Methods

### 2.1. Characterization and Maintenance of the Leishmania infantum Strain

For this study, the *Leishmania infantum* strain MHOM/BR/BH46 was used. It was maintained by cryopreservation in liquid nitrogen, using a preservation solution containing 10% dimethyl sulfoxide (DMSO) and 90% fetal bovine serum (FBS). For the experiments, the strain was reactivated and maintained in continuous culture in modified Schneider’s Drosophila medium, supplemented with 10% heat-inactivated fetal bovine serum (FBS), at a temperature of 26 °C, with subcultures performed every 72 h. The promastigote form was used for the in vitro assays, with growth observed and monitored through parasite counting using a Neubauer chamber (iNCYTO CChip DHC-N01, Cheonan-Si, Republic of Korea).

### 2.2. Synthesis of Indole-Thiosemicarbazone LTs Compounds

**Scheme 1:**
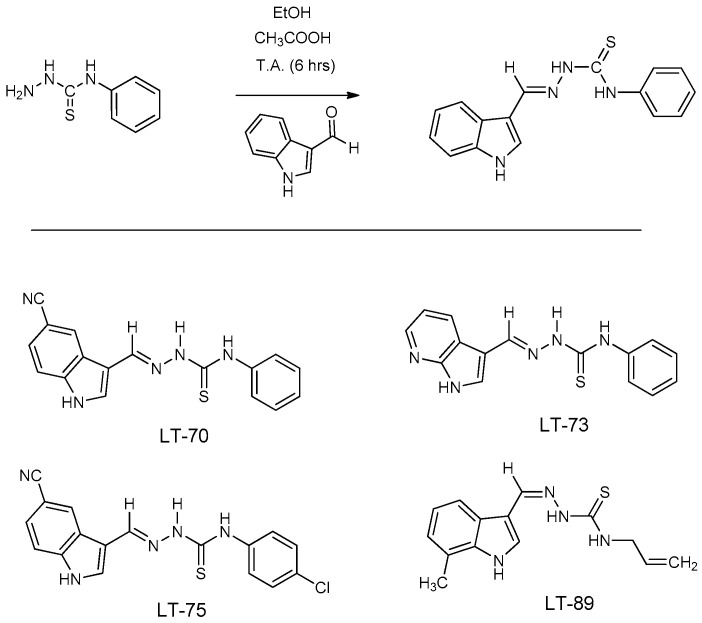
Chemical structure of indole-thiosemicarbazone compounds LTs.

The LT series compounds were synthesized at the Laboratory of Chemistry and Therapeutic Innovation (LQIT), Federal University of Pernambuco (UFPE), Recife, Brazil, following the protocol described by Jacob et al. [20] (Scheme 1). The synthesis was conducted in two main steps: (1) preparation of thiosemicarbazones through the reaction of hydrazine (80%, 2.0 mmol) with substituted isothiocyanates (1.0 mmol) in dichloromethane under magnetic stirring at room temperature; and (2) condensation of the resulting thiosemicarbazides with substituted indole-3-carboxaldehydes (1.0 mmol) under reflux conditions. The reactions were monitored by TLC, and the crude products were purified by recrystallization or column chromatography, yielding the final LT derivatives in satisfactory yields. Structural characterization, including ^1^H/^13^C NMR, FTIR, and melting point analysis, confirmed the purity and chemical identity of the compounds, consistent with the data previously reported by Jacob et al. [20].

### 2.3. In Vitro Evaluation of Leishmanicidal Activity of Thiosemicarbazone Compounds (LT-70, LT-73, LT-75, and LT-89)

The in vitro leishmanicidal activity of compounds LT-70, LT-73, LT-75, LT-89, and amphotericin B was determined as described by Aliança et al. [21], with adaptations. Promastigote forms of *L. infantum* (MHOM/BR/BH46) were maintained in Schneider’s medium supplemented with 10% fetal bovine serum (FBS), incubated at 26 °C. For experiments, parasites were subcultured and grown for 72 h until reaching stationary phase. Subsequently, they were adjusted to a concentration of 1 × 10^6^ parasites/mL. Cultures were then incubated in the presence of the tested compounds (20–400 µM) and Amphotericin B was tested from 0.01 to 1.0 µM in preliminary range-finding assays, confirming an IC_50_ of 0.04 µM, both prepared in DMSO (final DMSO concentration < 1%), as well as a control containing only the diluent. Analyses were performed in technical triplicate and biological duplicate. Cultures were maintained at 26 °C for 72 h, with daily parasite counts performed using a Neubauer chamber. The median inhibitory concentration (IC_50_) was determined after 72 h by linear regression using SPSS 25.0 software for Mac. All assays were performed in 24-well flat-bottom plates (2 mL final volume per well), with parasite inoculum verified by Neubauer counting chamber. The compounds LT-73 and LT-75 were selected based on their best leishmanicidal performance and respective selectivity index (SI), calculated as the ratio between the cytotoxic concentration for 50% of host cells (CC_50_) and the IC_50_ obtained for parasites. Compounds with higher SI were considered more selective and thus used in subsequent analyses. IC_50_ and CC_50_ values were obtained by linear regression using Microsoft Excel. This method has been validated in our previous thiosemicarbazone publications. Each assay was conducted in triplicate across two independent biological experiments performed on separate days to ensure reproducibility and minimize batch effects. MTT was selected for consistency with the validated thiosemicarbazone protocol by Jacob et al. [20], ensuring methodological coherence with prior data.

### 2.4. Differentiation of Promastigote Forms into Axenic Amastigote Forms

Differentiation of *L. infantum* promastigote forms into axenic amastigote forms was carried out according to the protocol by Oliveira et al. [22], with adaptations. Stationary-phase promastigotes (72 h) were cultured in modified Schneider’s Drosophila medium supplemented with 10% fetal bovine serum (FBS) at 26 °C. Cells were collected by centrifugation (3000× *g* for 10 min at 4 °C), washed three times with PBS (150 mM NaCl, 20 mM phosphate buffer, pH 7.2), and adjusted to 1 × 10^6^ cells/mL. Differentiation was induced in acidified Schneider’s medium (pH 5.5), supplemented with 2.5% FBS, incubated at 32 °C for seven days.

Axenic amastigotes were obtained from stationary-phase promastigotes of *L. infantum* following standard differentiation protocols. The cultures were maintained under controlled conditions and monitored daily by optical microscopy. Morphological observations confirmed the transition from elongated promastigotes to rounded amastigote-like forms with internalized or absent flagella. Morphological counting indicated that the majority of the culture (>90%) exhibited the amastigote phenotype before treatment. Cultures were regularly examined for microbial contamination, and no evidence of bacterial or fungal contamination was detected during the differentiation or experimental periods. Axenic amastigotes were exposed to compounds LT-73 and LT-75 at concentrations corresponding to the IC_50_ and 2× IC_50_, previously determined for promastigotes based on leishmanicidal activity, for analysis by scanning (SEM) and transmission electron microscopy (TEM).

### 2.5. Ultrastructural Analysis of L. infantum

Ultrastructural analysis of promastigote and axenic amastigote forms of *L. infantum* was performed as described by Silva et al. [16], with adaptations. Promastigote and amastigote forms were cultured in modified Schneider’s medium supplemented with 10% fetal bovine serum (FBS), in a BOD incubator at 26 °C and 32 °C, respectively, until reaching the stationary growth phase (72 h). After adjusting cell concentration to 1 × 10^7^ cells/mL, samples were incubated with compounds LT-73, LT-75, and amphotericin B at concentrations corresponding to the IC_50_ and 2× IC_50_ previously determined, as well as a DMSO control (1% diluted), for 72 h. During incubation, daily microscopic monitoring of cell morphology was performed.

After this period, cells were washed with PBS (phosphate-buffered saline) and fixed in a solution containing 2.5% glutaraldehyde and 4% formaldehyde in 0.1 M phosphate buffer (pH 7.4) for 24 h at 4 °C. For transmission electron microscopy (TEM), cells were post-fixed in 1% osmium tetroxide (OsO_4_) for 2 h in 0.1 M phosphate buffer (pH 7.2), dehydrated in increasing acetone series (30%, 50%, 70%, 90%, and 3× 100%), embedded in EPON resin, and subjected to ultrathin sectioning using an ultramicrotome. Sections were contrasted with 2% uranyl acetate for 30 min and 2.5% lead citrate for 10 min, then analyzed using a Tecnai G2 Spirit TEM (FEI).

For scanning electron microscopy (SEM), after primary fixation, cells were adhered to coverslips pre-treated with poly-L-lysine for 30 min. Then, cells were washed three times with 0.1 M phosphate buffer (pH 7.2), post-fixed in 1% OsO_4_ for 2 h, dehydrated in increasing ethanol series (30%, 50%, 70%, 90%, and 3× 100%), subjected to critical point drying using an HCP-2 device (Hitachi (Tokyo, Japan)), and gold-coated (20 nm) using a JFC-1100 device ((JEOL Ltd., Tokyo, Japan)). Visualization was performed using a JEOL JSM-5600LV (JEOL Ltd., Tokyo, Japan) scanning electron microscope.

All images were acquired under standardized laboratory protocols. A minimum of 50 parasites per condition were analyzed by two blinded investigators and no visible precipitation or crystallization occurred during fixation.

### 2.6. Cytokine Dosage of the Cellular Immune Response After Treatment of LT-73 and LT-75 Compounds

J774.A1 macrophage lineage cells were cultured in 96-well polystyrene plates containing DMEM medium supplemented with 10% FBS. Cells were incubated with the following concentrations: 1× CC_50_ and 2× CC_50_ of compounds LT-73 and LT-75 and amphotericin B, as well as 1% DMSO diluent solution as control, for 72 h. The cells used in these assays were not infected with *Leishmania infantum*, allowing the evaluation of cytokine modulation and cytotoxicity in uninfected macrophages. After cultivation, 100 µL of culture supernatants were collected and immediately stored at −20 °C for later use in CBA assays (Cytometric Bead Array-BD Biosciences, BD Biosciences, San Jose, CA, USA). This assay utilized a mixture of 6 polystyrene beads with different fluorescence intensities, coated with specific antibodies for quantification of cytokines IFN-γ, TNF, IL-10, IL-6, IL-4, IL-2, and IL-17a present in sample supernatants. Cytokine levels were quantified using the BD Cytometric Bead Array (CBA) Mouse Th1/Th2/Th17 kit (Cat. No. 560485, BD Biosciences, San Jose, CA, USA) and analyzed on a BD FACSCalibur™ Flow Cytometer with BD CellQuest PRO Software (BD Biosciences; version 5.1 by the facility). Results are expressed in pg/mL based on standard curves provided by the manufacturer.

### 2.7. Evaluation of Mitochondrial Membrane Potential (ΔΨm)

To evaluate the effects of compounds on the mitochondrial membrane potential (ΔΨm) of *Leishmania infantum*, the cationic probe Rhodamine 123 (Rod 123) was used through quantitative flow cytometry analysis. Promastigote form parasites of *L. infantum* strain (MHOM/BR/BH46) were cultivated in Schneider’s medium supplemented with 10% fetal bovine serum (FBS), maintained at 26 °C for 72 h until reaching stationary phase. Cells were adjusted to a concentration of 1 × 10^6^ promastigotes/mL and incubated with compounds LT-73 and LT-75, selected based on previously observed best selectivity indices. Tested concentrations were 1× IC_50_ and 2× IC_50_ for 72 h under the same conditions used in leishmanicidal activity assays. After incubation, cells were centrifuged at 3000× *g* for 5 min, and supernatants discarded. Afterward, the treated and untreated samples were incubated in 2 μg/mL of Rhodamine 123 (Rho 123) for 30 min and washed three times in 1× PBS. Hydrogen peroxide (H_2_O_2_) at 1 μM was used as positive control, previously described in the literature as an inducer of mitochondrial potential loss. Cells were analyzed by flow cytometry on the FACSCalibur equipment (Becton & Dickinson, San José, CA, USA), with excitation at 488 nm and detection at 505/534 nm. Fifty thousand events per sample were acquired. Data were expressed as percentage of cells stained with Rhodamine 123 for each tested condition. Fluorescence variation was quantified by Variation Index (VI), calculated by the equation: VI = (MT − MC)/MC, where MT corresponds to the median fluorescence of treated cells and MC to the median of control cells (untreated).

### 2.8. Evaluation of Apoptosis and Necrosis in L. infantum Promastigote Forms

To evaluate cell death types induced by the compounds, promastigote forms of *Leishmania infantum* (2 × 10^6^ cells/mL) were treated with concentrations corresponding to 1× and 2× IC_50_ of compounds LT-73 and LT-75 for 72 h. After incubation, cells were centrifuged at 3000× *g* for 5 min, washed with PBS, and subsequently with 1× Buffer Solution provided by the Annexin V-FITC apoptosis detection kit (BD Biosciences, San Jose, CA, USA). Cells were then resuspended in 100 µL Buffer Solution and stained with 5 µL Annexin V conjugated to FITC, incubated for 15 min at room temperature protected from light. After incubation, cells were washed again with 1× Buffer Solution and resuspended in 200 µL of the same buffer. Subsequently, 5 µL propidium iodide (PI) were added to each sample for identification of cells with compromised membrane integrity. Samples were transferred to flow cytometry tubes and analyzed on the FACSCalibur flow cytometer (Becton Dickinson, San José, CA, USA) equipped with CellQuest PRO Software (BD Biosciences; version 5.1), within 4 h after staining. Annexin V-FITC was excited at 488 nm, with fluorescence detected at 530/30 nm; propidium iodide (PI) was excited at 488 nm and detected at 585/42 nm. Single-stained controls for Annexin V-FITC and PI were included in order to apply spillover correction (compensation) between channels.

### 2.9. Statistical Analysis

All experiments were performed in at least three independent replicates. Data are expressed as mean ± standard deviation (SD). One-way analysis of variance (ANOVA) followed by Tukey’s post hoc test was used for multiple group comparisons, and differences with *p* < 0.05 were considered statistically significant. Dose–response curves for the determination of IC_50_ and CC_50_ values were fitted by linear regression using GraphPad Prism 10.0 (GraphPad Software, San Diego, CA, USA).

## 3. Results

### 3.1. Leishmanicidal Activity of LT Compounds

The LT compounds demonstrated strong leishmanicidal activity, with IC_50_ values ranging from 10.5 to 14 µM, reaching almost 100% inhibition even at the lowest concentrations tested (20 to 400 µM). Amphotericin B, used as a positive control, presented an IC_50_ of 0.04 µM, showing significant inhibition even at low concentrations.

Table 1 presents the mean IC_50_ values obtained for *Leishmania infantum* promastigote forms, the CC_50_ values for J774.A1 macrophage lineage, and the corresponding selectivity indices (SI) of compounds LT-70, LT-73, LT-75, LT-89, and amphotericin B.

The cytotoxicity of the LT compounds, assessed by determination of CC_50_, ranged from 60 µM to over 75 µM. Compounds LT-73 and LT-75 did not exhibit significant cytotoxicity up to the highest concentration tested (CC_50_ > 75 µM). Amphotericin B, in turn, presented a CC_50_ of 14 µM, indicating greater toxicity toward macrophages compared to the tested compounds.

The selectivity index (SI), calculated as the ratio between CC_50_ and IC_50_, ranged from 4.3 to >7.1 among the LT compounds. Amphotericin B displayed a markedly higher SI (350) compared to the LT derivatives (SI range 4.3–>7.1). We therefore avoid claiming superior selectivity for the LTs; rather, we consider these LTs as lead candidates that require further optimization to approach the potency/selectivity of current drugs.

All IC_50_ and CC_50_ determinations were performed in duplicate biological assays, each with technical replicates. Mean values are reported with standard deviation to reflect reproducibility. The CC_50_ values marked as ‘>75 µM’ correspond to the highest concentration tested, indicating that cytotoxicity was not observed within this range. Estimated SI values for axenic amastigotes were calculated using the IC_50_ values obtained for the promastigote forms.

### 3.2. Ultrastructural Analysis of L. infantum Promastigote Forms

Promastigote and axenic amastigote forms of *L. infantum*, treated or not with compounds LT-73, LT-75, and Amphotericin B, were evaluated by electron microscopy to investigate morphological and ultrastructural changes induced by the treatments.

#### 3.2.1. *L. infantum* Promastigote Forms

**Figure 1:**
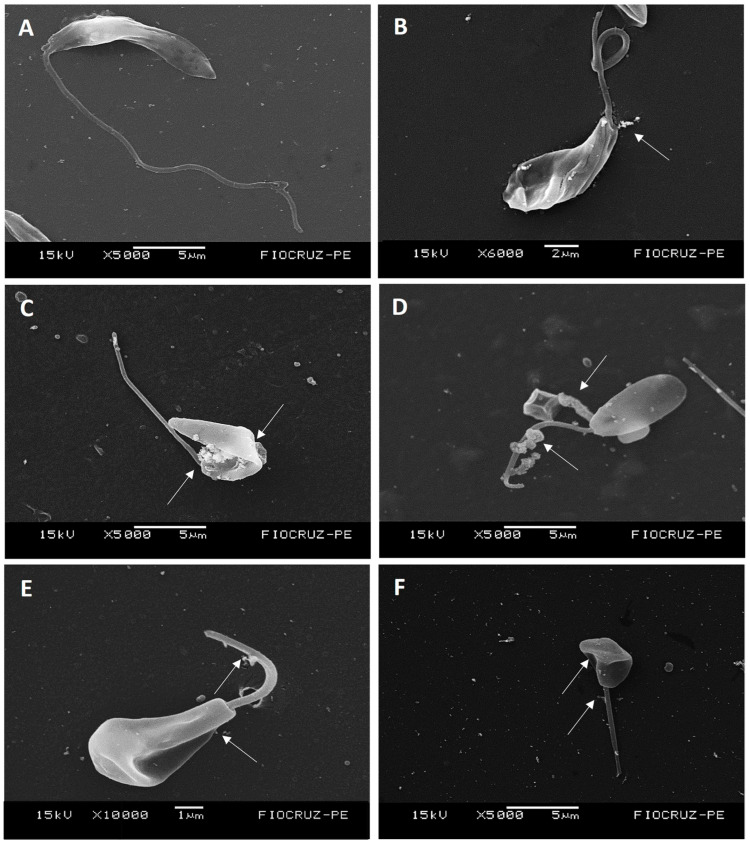
(**A**–**F**) Electron micrographs of untreated *L. infantum* promastigote forms and those treated with the heterocyclic compounds LT-73, LT-75, and Amphotericin B: (**A**) Control cells treated with 1% DMSO, showing a fusiform shape with preserved surface and elongated flagellum. (**B**) Cells exposed to 1× IC_50_ of Amphotericin B, displaying membrane rupture and leakage of cytoplasmic contents (long white arrow). (**C**,**D**) Cells treated with LT-73 at 1× IC_50_ and 2× IC_50_, respectively. Note increased cell size, membrane invagination with rupture, and loss of cytoplasmic contents. (**E**,**F**) Cells treated with LT-75 at 1× IC_50_ and 2× IC_50_, respectively. Note increased cell size, altered morphology, membrane invagination with rupture, and loss of cytoplasmic contents, no visible precipitation or crystallization occurred during fixation. The images were obtained with a magnification between 5000 and 10,000, and the scale bar ranged from 1 to 5 µm, as can be observed below each image.

In the SEM analyses, promastigotes from the control group exhibited typical fusiform morphology with preserved flagella (Figure 1A). In contrast, cells treated with Amphotericin B (1× and 2× IC_50_) showed membrane rupture and leakage of cytoplasmic content (Figure 1B). Similar morphological alterations were observed following treatment with LT-73 (1× and 2× IC_50_), including increased cell volume, membrane invagination, rupture, and loss of cytoplasmic content (Figure 1C,D). Treatment with LT-75 (1× and 2× IC_50_) induced cell enlargement and changes in overall cell body morphology, with disorganization of the plasma membrane and leakage of cytoplasmic content (Figure 1E,F).

**Figure 2:**
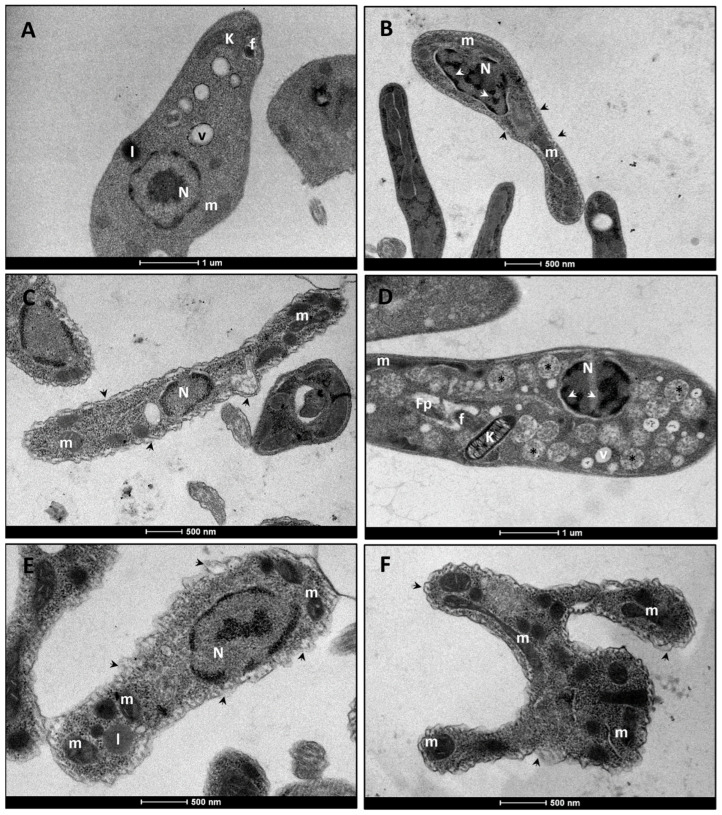
(**A**–**F**) Electron micrograph of *L. infantum* promastigotes, untreated and treated with the heterocyclic compounds (LT-73 and LT-75) and Amphotericin B. (**A**) Control cells treated with 1% DMSO. Note the fusiform morphology and preservation of all internal structures, including normal mitochondria (m). (**B**) Cells treated with 1× IC_50_ of Amphotericin B. Observe membrane rupture (black arrowhead), chromatin condensation (white arrowhead), and mitochondrial swelling (m). (**C**,**D**) Cells treated with LT-73 at 1× and 2× IC_50_, respectively. Note morphological alterations with plasma membrane disorganization (short arrow), chromatin condensation (white arrowhead), mitochondrial swelling (m), presence of vacuoles (V), and multiple vacuoles containing dispersed granular, circular, and electrondense material in the cytoplasm (asterisks). (**E**,**F**) Cells treated with LT-75 at 1× and 2× IC_50_, respectively. Observe severe disorganization of cell morphology, with extensive damage to the plasma membrane and cytoskeleton (black arrowhead), leading to disruption of internal structures. Nucleus (N), vacuole (v), mitochondrion (m, swollen in treated cells), Kinetoplast (K), lipid inclusion (l), flagellum (f), flagellar pocket (fp). The scale bar ranged from 500 nm to 1 µm, as can be observed below each image.

TEM analyses confirmed the alterations observed by SEM. Control promastigotes exhibited fusiform morphology and preserved internal structures, such as the nucleus, mitochondrion, and kinetoplast (Figure 2A). Exposure to Amphotericin B (1× IC_50_) induced membrane rupture, chromatin condensation, and mitochondrial swelling (Figure 2B). Compound LT-73 (1× and 2× IC_50_) caused disorganization of the plasma membrane, mitochondrial swelling, chromatin condensation, and the presence of numerous vacuoles containing granular and electron-dense material dispersed throughout the cytoplasm (Figure 2C,D). In turn, LT-75 (1× and 2× IC_50_) promoted extensive damage to the plasma membrane and cytoskeleton, compromising the integrity of internal structures, with emphasis on severe alterations in the mitochondrion (Figure 2E,F).

#### 3.2.2. *L. infantum* Amastigote Forms

**Figure 3:**
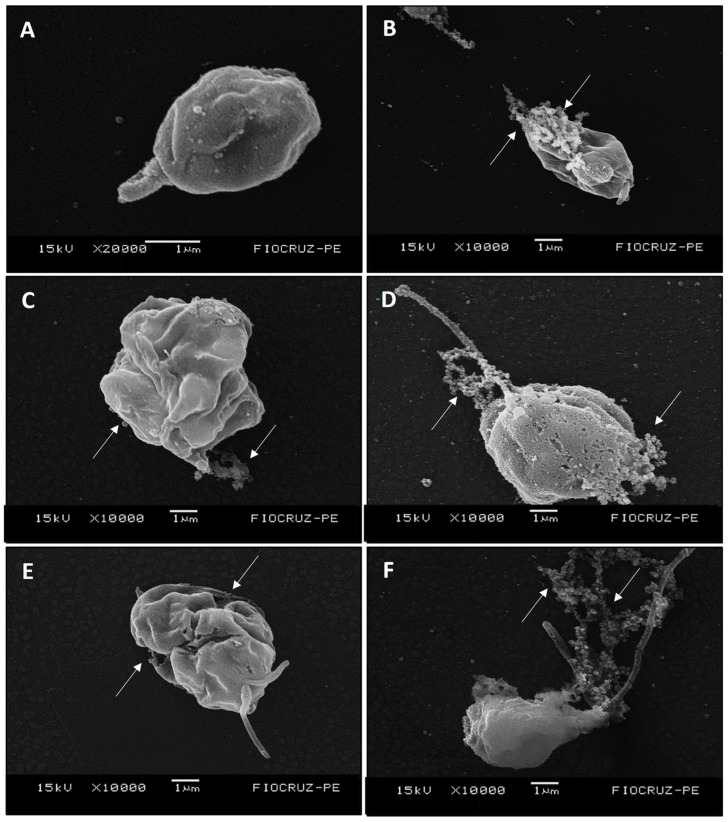
(**A**–**F**) Electron micrographs of *L. infantum* axenic amastigotes, untreated and treated with the heterocyclic compounds (LT-73 and LT-75) and Amphotericin B. (**A**) Control cells treated with 1% DMSO, exhibiting an oval body, short flagellum, and preserved morphology. (**B**) Cells treated with 1× IC_50_ of Amphotericin B, showing membrane rupture and cytoplasmic content leakage (long white arrow). (**C**,**D**) Cells treated with LT-73 at 1× and 2× IC_50_, respectively, displaying increased cell size, membrane invagination with rupture, and loss of cytoplasmic content. (**E**,**F**) Cells treated with LT-75 at 1× and 2× IC_50_, respectively, showing increased cell size, membrane invagination with rupture, loss of cytoplasmic content, and flagellum duplication. The images were obtained with a magnification between 10,000 and 20,000, and the scale bar corresponds to 1 µm, as can be observed below each image.

Untreated amastigotes analyzed by scanning electron microscopy exhibited oval morphology and short flagellum, with preserved morphological integrity (Figure 3A). Treatment with Amphotericin B (1× IC_50_) induced membrane rupture and leakage of cytoplasmic content (Figure 3B). Similar alterations were observed following treatment with LT-73 (1× and 2× IC_50_), such as increased cell volume, membrane invagination and rupture, and loss of cytoplasmic content (Figure 3C,D). Amastigotes treated with LT-75 (1× and 2× IC_50_) also exhibited increased cell volume, marked morphological alterations, and membrane rupture with cytoplasmic leakage (Figure 3E,F).

**Figure 4:**
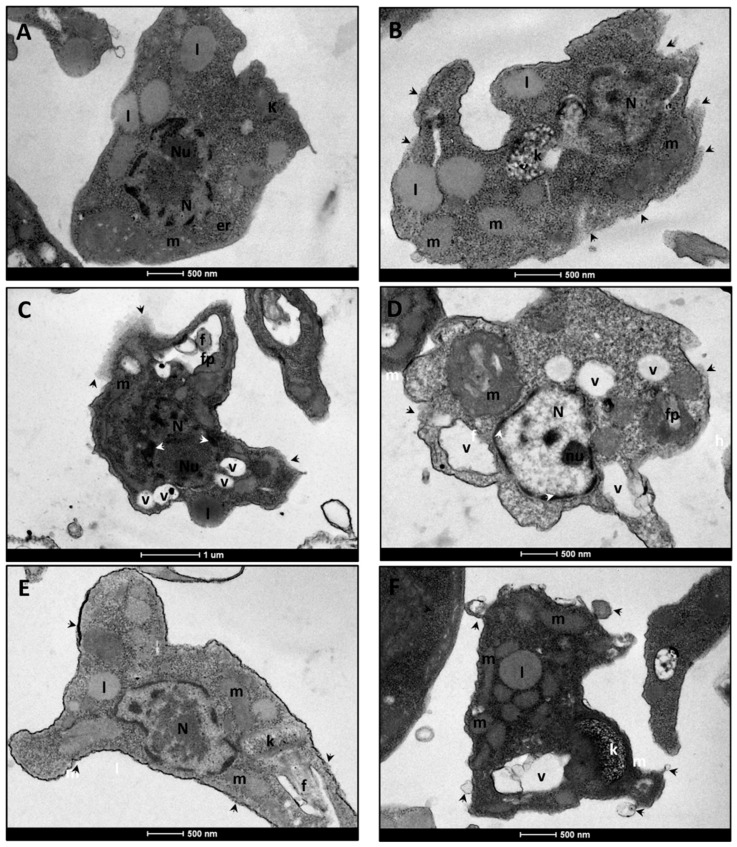
Electron micrographs of untreated *L. infantum* axenic amastigotes forms and those treated with the heterocyclic compounds LT-73, LT-75, and Amphotericin B. (**A**) Control cells treated with 1% DMSO. The preserved cell body and internal structures morphology. Note nucleus (N), nucleolus (Nu), kinetoplast (k), endoplasmic reticulum (er), lipid (l) and mitochondria (m). (**B**) Cells treated with 1× IC_50_ of Amphotericin B, showing plasma membrane rupture (black arrowhead), altered kinetoplast morphology (k), and miResulttochondrial swelling (m). (**C**,**D**) Cells treated with LT-73 at 1× and 2× IC_50_, respectively. Note altered cell morphology with disarrangement of the plasma membrane (black arrowhead) and flagellar pocket (fp), chromatin condensation (white arrowhead), mitochondrial swelling (m), and increased number of vacuoles (v). (**E**,**F**) Cells treated with LT-75 at 1× and 2× IC_50_, respectively. Cells exhibit severe damage to the plasma membrane and cytoskeleton (black arrowhead), causing disorganization of cell morphology and, consequently, internal structures, particularly mitochondrial (m, swollen in treated cells) and kinetoplast (k) swelling. The scale bar ranged from 500 nm to 1 µm, as can be observed below each image.

In TEM, control group amastigotes presented a preserved nucleus and organelles, maintaining intact cellular morphology (Figure 4A). Amphotericin B (1× IC_50_) promoted plasma membrane rupture, alterations in the kinetoplast, and mitochondrial swelling (Figure 4B). Treatment with LT-73 (1× and 2× IC_50_) induced significant alterations in the plasma membrane and flagellar pocket, chromatin condensation, mitochondrial swelling, and an increased number of intracellular vacuoles (Figure 4C,D). LT-75 (1× and 2× IC_50_) caused severe damage to the plasma membrane and cytoskeleton, disrupting cellular morphology and compromising internal structures, especially the mitochondrion and kinetoplast (Figure 4E,F).

Therefore, the ultrastructural changes observed, including plasma membrane disorganization and cytoplasmic vacuolization, are compatible with late apoptotic events, although a direct effect of the compounds on these structures cannot be excluded.

### 3.3. Results of Cytokine Dosage of the Cellular Immune Response After Treatment of LT-73 and LT-75 Compounds

The immunomodulatory profile analysis was performed using the supernatants from J774.A1 macrophage cultures stimulated for 72 h with 1× and 2× CC_50_ concentrations of the heterocyclic compounds LT-73, LT-75, and Amphotericin B.

**Figure 5:**
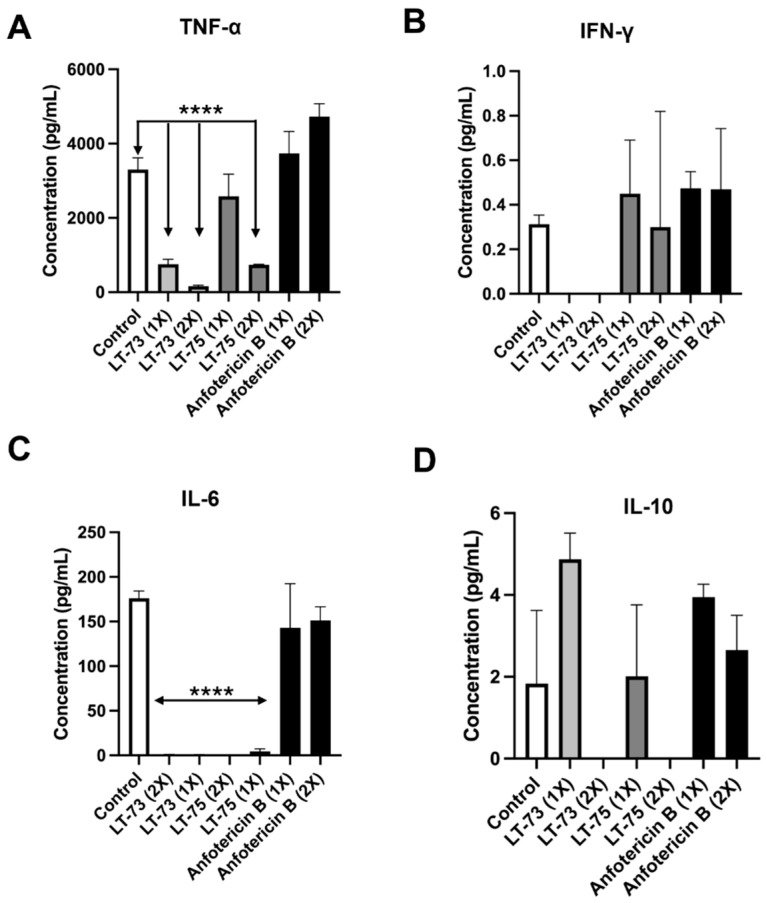
Induction of cytokine production from cellular immune responses Th1, Th2, and Th17. (**A**) TNF-α cytokine levels. (**B**) IFN-γ cytokine levels. (**C**) IL-6 cytokine levels. (**D**) IL-10 cytokine levels. Control = no-treatment and **** (*p* ≤ 0.0001).

Analysis of TNF-α production revealed that compounds LT-73 and LT-75 induced the production of this cytokine at the lowest concentrations tested (Figure 5A). However, the 1× and 2× CC_50_ concentrations of LT-73 and LT-75 showed a significant reduction in TNF-α production compared to the control. The LT-75 1× and 2× CC_50_ induced the production of the cytokine IFN-γ, both in relation to the compounds themselves and in comparison, with Amphotericin B treatments (Figure 5B), while no cytokine production was observed for both LT-73 concentrations.

Both compounds induced a significant reduction in IL-6 secretion compared to the untreated control and Amphotericin B. Only a minimal production in IL-6 was observed, with a discrete elevation detected at 1× LT-75 (Figure 5C). Both compounds also induced the production of the cytokine IL-10 at the 1× CC_50_ concentration at the tested time point. However, at the 2× CC_50_ concentration, a reduction in IL-10 production was observed (Figure 5D). No production of IL-2, IL-4, or IL-17A was observed at the tested concentrations.

### 3.4. Cell Death Analysis

#### 3.4.1. Annexin and Propidium Iodide

Flow cytometry analysis, using double staining with annexin V (AV) and propidium iodide (PI), enabled the identification of different cell death profiles in *L. infantum* promastigotes treated with LT-73, LT-75, and Amphotericin B for 72 h.

**Figure 6:**
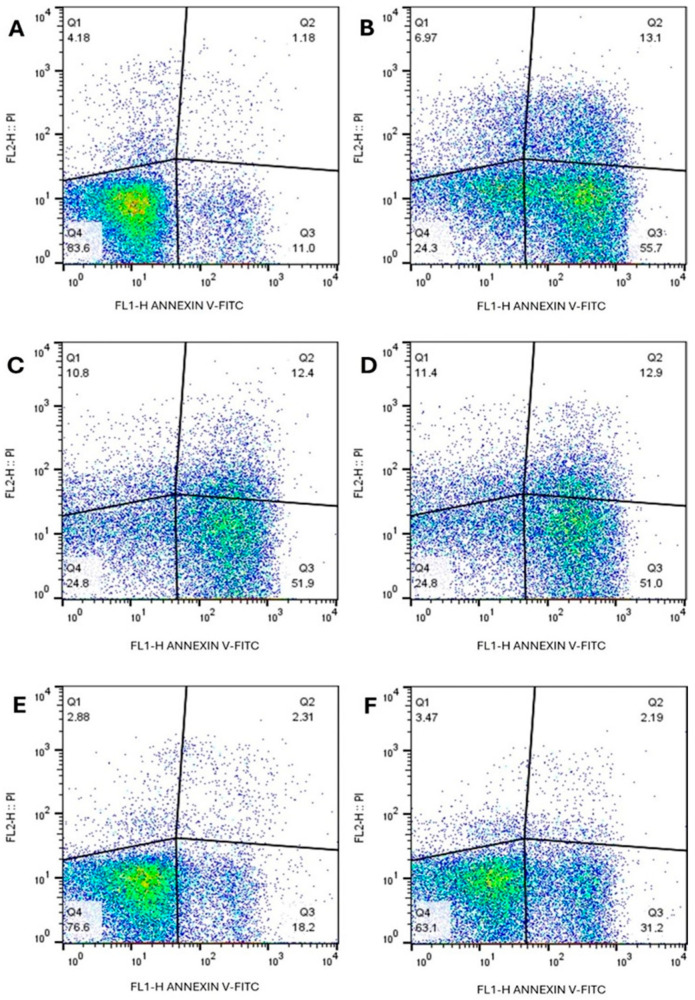
Evaluation of phosphatidylserine exposure and plasma membrane integrity in *L. infantum* promastigotes treated with compounds LT-73, LT-75, and Amphotericin B, assessed by flow cytometry (**A**) Control cells, untreated. (**B**) Amphotericin B 2× IC_50_. (**C**) LT-73 1× IC_50_, (**D**) LT-73 2× IC_50_, (**E**) LT-75 1× IC_50_ and (**F**) LT-75 2× IC_50_ compounds.

Treatment with LT-73 predominantly induced AV+/PI− staining, indicative of early apoptotic events, together with a smaller proportion of double-positive cells (AV+/PI+), consistent with apoptosis-like processes often described as ‘late apoptosis’ in *Leishmania* studies, though this pattern may also reflect membrane permeabilization associated with necrotic cell death. Only a minor population of AV−/PI+ cells was detected, suggestive of necrotic cell death through plasma membrane disruption, under both 1× and 2× IC_50_ (Figure 6C,D). Similarly, LT-75 treatment was characterized by predominance of AV+/PI− staining, particularly at the 2× IC_50_ concentration, thereby strengthening the evidence that this compound also promotes an apoptosis-like mode of cell death (Figure 6E,F).

Amphotericin B, used as a positive control, also induced apoptosis as the main form of cell death, with higher percentages than those observed for the tested compounds. On the other hand, untreated control promastigotes exhibited low staining for AV and PI, confirming cell viability.

These results, shown in Figure 6, demonstrate that compounds LT-73 and LT-75 predominantly induced apoptosis in *L. infantum* promastigotes, with a smaller proportion of necrotic cells induced by LT-73 treatment.

#### 3.4.2. Assessment of Membrane Potential

The mean fluorescence values of the compounds ranged from 213 to 1425, while the Variability Index (VI) fluctuated between 1 and −0.73 (Table 2). The VI for treatment with 1× and 2× IC_50_ of LT-73 ranged from −0.02 to −0.07, indicating depolarization of the mitochondrial membrane at these concentrations. However, at 1× and 2× IC_50_ concentrations of LT-75, mitochondrial membrane depolarization was not observed. Nevertheless, amphotericin B and hydrogen peroxide (H_2_O_2_) showed the most significant VI values, ranging from −0.018 to −0.73.

## 4. Discussion

The present study demonstrates that the thiosemicarbazone derivatives LT-73 and LT-75 display strong and selective leishmanicidal activity against both promastigote and axenic amastigote forms of *Leishmania infantum*, while showing low cytotoxicity toward mammalian macrophages. The ultrastructural and immunological analyses revealed that these effects are associated with apoptosis-like cell death and mitochondrial dysfunction, supporting the potential of these compounds as selective antileishmanial candidates [20,23].

The chemical characterization of these compounds, previously reported by Jacob et al. [20], confirmed the thione tautomeric form and the expected structural integrity, supporting their suitability for biological evaluation. All thiosemicarbazone compounds investigated and described in this work were tested for their leishmanicidal activity. The promastigotes cells were used as an initial screening model and axenic amastigotes as a closer approximation to the clinical stage. This model, widely used for decades, provides high reproducibility and scalability for pharmacological assays, supporting the antileishmanial activity observed for the tested compounds [24]. The LT-73 and LT-75 treatment showed the best results regarding inhibitory concentration (IC_50_) and selectivity index (SI). These molecules contain a phenyl group, radicals that contribute to the reactivity of the molecules, which may alter their stability, thereby contributing to their leishmanicidal activity. The presence of these radicals was also observed in the molecules used by Queiroz et al. [23], who also evaluated the efficacy of others thiosemicarbazone compounds against promastigote and amastigote forms of *Leishmania infantum*. Aliança et al. [21] also evaluated the leishmanicidal action of compounds that likewise had a phenyl radical in their molecule, as well as a methoxy radical, which potentiated the leishmanicidal activity in promastigote forms of *L. infantum*. However, the compounds used in this study were never tested before against promastigote and amastigote forms of *L. infantum*.

The cytotoxic action of LT-70, LT-73, LT-75, LT-89 and amphotericin B treatment were also evaluated in J774 macrophages, previously reported by Jacob et al. [20], reinforcing the low cytotoxicity profile of these compounds in assays performed within our research group, with LT-73 and LT-75 displaying the best cytotoxicity and selectivity index results and also being less toxic than amphotericin B in absolute values. Similar results were found in the study by Aliança et al. [21], who also evaluated the cytotoxicity of heterocyclic compounds 2j (362.6 µM) and 2m (263.6 µM) from the thiazole class in J774 macrophages and Vero cells, which were also less toxic than amphotericin B (74.7 µM). The lower toxicity of heterocyclic compounds was also observed in the work by Queiroz et al. [23], who evaluated the cytotoxicity of compounds JW-16.2 (362.65 µM) and GT-14 (635.33 µM) in peritoneal macrophages compared to amphotericin B (74.86 µM).

Although amphotericin B demonstrates superior in vitro selectivity in our assays, the LT compounds may offer complementary advantages such as chemical tractability for medicinal chemistry optimization, potential differences in formulation or administration, and lower synthetic cost [25,26,27,28]. These potential advantages remain speculative and require pharmacokinetic and in vivo evaluation; therefore, our conclusions focus on identifying LT-73 and LT-75 as preliminary lead structures rather than clinical alternatives to amphotericin B. When compared with reference antileishmanial drugs, the IC_50_ values of LT-73 and LT-75 (10–14 µM) fall within the same order of magnitude as those described for miltefosine against *Leishmania infantum* (≈8–12 µM under similar in vitro conditions) [25,29]. Despite miltefosine exhibiting comparable potency, its gastrointestinal toxicity and teratogenic potential remain major clinical limitations. Conversely, LT-73 and LT-75 demonstrated low cytotoxicity toward macrophages (CC_50_ > 75 µM), suggesting a more favorable selectivity profile in vitro. Moreover, considering the increasing interest in combination therapy as a strategy to enhance efficacy and reduce resistance, it is noteworthy that synergistic or additive effects have been reported for miltefosine in association with amphotericin B or other small molecules [9,10]. Although combination studies were not included in the present work, the dual mitochondrial and immunomodulatory effects of LT-73 and LT-75 highlight their potential for future evaluation in combination regimens.

The LT-73 and LT-75 compounds exhibited selectivity index ranging from 3.8 to 4.5, indicating moderate selectivity toward *Leishmania infantum*. Compounds with SI values above 2 are considered biologically relevant in preliminary in vitro studies, as they demonstrate lower cytotoxicity to mammalian cells while maintaining antiparasitic efficacy [23,30,31]. Although these values are below the ideal threshold (SI ≥ 10) proposed for preclinical candidates [32,33], they are comparable to those reported for other thiosemicarbazone derivatives, supporting LT-73 and LT-75 as promising scaffolds for further optimization of potency and safety profiles.

Ultrastructural analysis by SEM of *L. infantum* promastigote forms demonstrated that LT-73 and LT-75 induced alterations including cell enlargement, membrane invagination with rupture and loss of cytoplasmic content, as well as flagellar shrinkage. Similar results were found in the studies of Queiroz et al. [23], Aliança et al. [21], and Silva et al. [16], which demonstrated the same ultrastructural changes induced by the tested compounds. The ultrastructural alterations caused by exposure of promastigote forms to LT-73 and LT-75 described in this study may be related to the production of reactive oxygen species (ROS), which can indirectly and directly affect macromolecule production [34]. Although previous studies have reported the antileishmanial potential of indol-3-yl-thiosemicarbazone derivatives [16], our study extends this knowledge by investigating structurally distinct thiosemicarbazone analogs (LT-70, LT-73, LT-75, and LT-89), which differ in their aromatic substitutions and electronic properties. These molecular modifications may alter their biological reactivity and selectivity toward parasite targets. Furthermore, the present work incorporates a broader biological evaluation, combining leishmanicidal and cytotoxicity assays with ultrastructural and immunomodulatory analyses to provide new insights into the mechanisms underlying the antiparasitic effects of this chemical class.

Ultrastructural analysis by TEM of *L. infantum* promastigote forms exposed to LT-73 and LT-75 showed alterations such as membrane rupture, chromatin condensation, mitochondrial swelling, and cellular disorganization, as well as cytoplasmic vacuoles containing electron-dense granular material, compromising the membrane, cytoskeleton, and internal structures. Similar results were observed in the studies by Silva et al. [16] and Gouveia et al. [35], evaluating thiosemicarbazone and thiazolidinic compounds, respectively, in *L. infantum* promastigotes. These studies reported mitochondrial swelling, presence of electron-dense particles, and vacuoles. According to Kaczanowski et al. [36], the ultrastructural changes found in this study are compatible with cell death by apoptosis.

The molecules that showed the best leishmanicidal results in the promastigote forms were also tested in the amastigote forms. Ultrastructural analysis by SEM of axenic amastigote forms of *L. infantum* revealed significant alterations, including membrane rupture with cytoplasmic leakage and cell size reduction. These alterations were similar to those observed in promastigote forms, which according to Minori et al. [37], may be related to mitochondrial oxidative stress that can increase or decrease mitochondrial membrane potential, leading to the apoptotic process. Plasma membrane lysis was also observed by Oliveira et al. [22], who treated axenic amastigote forms of *Leishmania amazonensis* with the IC_50_ (13.5 μM) of the Calpain Inhibitor (MDL28170), a reversible peptidomimetic inhibitor.

Through TEM analysis of axenic amastigote forms of *L. infantum* treated with compounds LT-73 and LT-75, it was possible to observe plasma membrane disorganization, chromatin condensation, mitochondrial swelling, and an increase in the number of vacuoles. The same alterations were found in the promastigote forms tested in this study when subjected to LT-73 and LT-75, indicating that the tested compounds present similar effects regardless of the parasitic form of *L. infantum*. These alterations, previously reported by Tiuman et al. [38], were considered indicative of a cell death process, inducing cells to recycle cytoplasmic components to maintain their survival.

Understanding the interaction of compounds with the immune system in leishmaniasis has been highlighted in several studies, as evidenced by Kolodziej & Kiderlen [39]. According to Rodrigues et al. [40], the immunopathogenesis of the disease depends on the balance between Th1 and Th2 profiles, with Th1 profile cytokines being associated with resistance to *Leishmania* infection, while Th2 cytokines are linked to susceptibility to parasitic infection. In this study, cytokine assays were performed using uninfected J774.A1 macrophages treated with the compounds to evaluate their direct immunomodulatory potential independent of parasite infection. Compounds LT-73 and LT-75, at concentrations of 1× and 2× the CC_50_ in J774 macrophages, were able to induce the production of TNF-α, IFN-γ, and IL-10. TNF-α together with IFN-γ are produced by macrophages after pathogen recognition and may increase NO production and control parasitemia levels, according to Silva et al. [41]. IL-6 production was observed only at the CC_50_ concentration of LT-73 and 1× CC_50_ of LT-75. According to Conceição et al. [42], compounds that increase or decrease IL-6 and IL-10 production may be associated with more intense activity against trypanosomatids, as they are capable of inducing both pro-inflammatory and regulatory molecules, thus contributing to the reduction in the exacerbation of the immune response [43]. Similar results were observed by Jacob et al. [20], who evaluated the compounds LT-76, LT-81, and LT-87 regarding their anti-inflammatory activity, compounds from the same class used in this study.

There are several types of cell death, one of which is apoptosis, already observed in various unicellular eukaryotes, including species of the genus *Leishmania* [44]. According to Santos et al. [15], phosphatidylserine externalization favors the identification of apoptotic cells. In the present study, LT-73 and LT-75 at 1× and 2× IC_50_ concentrations were able to induce phosphatidylserine externalization in promastigote forms of *L. infantum* after 72 h of treatment; the same behavior was observed in cells treated with amphotericin B. Concomitant labeling with Annexin V and propidium iodide was also observed in LT-73 and amphotericin B treated cells, indicating altered membrane permeability that allowed double staining and suggesting a late apoptosis-like process. In *Leishmania*, this pattern is often interpreted as apoptosis-like cell death associated with membrane permeabilization, though necrotic features cannot be excluded [29]. While apoptosis-like events appeared predominant, PI-only labeling also indicated necrotic processes in a smaller fraction of parasites under LT-73 and amphotericin B treatment, suggesting that higher concentrations or prolonged exposure may further compromise membrane integrity.

Mitochondria play a central role in cell death mechanisms in trypanosomatids, including species of the genus *Leishmania*. The alteration of mitochondrial membrane potential (Δψm) is a key indicator of apoptotic or necrotic processes [23,45]. In this study, we observed that LT-73 and LT-75 induced a alteration in Δψm, suggesting a potential to induce reactive oxygen species (ROS) production, mitochondrial swelling, and chromatin alterations, as evidenced in our TEM analyses. Although mitochondrial depolarization suggests potential ROS involvement, further studies using DCFDA or MitoSOX assays are needed to confirm oxidative stress mechanisms. Similar results were reported by Queiroz et al. [23], who also observed disruption of membrane potential in *L. infantum* after treatment with compounds JW-16.2 and GT-14. These findings reinforce the crucial role of mitochondria as therapeutic targets in trypanosomatids.

Although the present in vitro and ultrastructural results provide robust evidence of leishmanicidal activity and mitochondrial disruption by LT derivatives, this study has some limitations inherent to early-stage screening. All assays were performed on promastigote and axenic amastigote forms of *Leishmania infantum*, without the inclusion of intracellular infection models. Future studies will therefore employ infected macrophage systems (e.g., RAW 264.7 or THP-1–derived cells) to confirm the intracellular efficacy and selectivity of the most active compounds (LT-73 and LT-75). In addition, in vivo investigations will be necessary to evaluate pharmacokinetic parameters, systemic toxicity, and therapeutic efficacy. These steps will be essential to validate the translational potential of LT derivatives and support their further preclinical development.

## 5. Conclusions

The search for new therapeutic agents for Visceral Leishmaniasis requires compounds that combine safety, accessibility, and proven efficacy, along with well-defined pharmacological properties. In this study, thiosemicarbazone derivatives LT-73 and LT-75 demonstrated promising leishmanicidal activity against promastigote and amastigote forms of *Leishmania infantum*, associated with apoptotic-like cell death mechanisms. Although these findings provide encouraging evidence of biological activity and selectivity, further research is required to confirm their therapeutic potential. Future studies will include assays using infected macrophage models to evaluate intracellular efficacy, as well as in vivo investigations to assess pharmacokinetics, systemic toxicity, and overall safety profiles.

## Figures and Tables

**Table 1:** Results of leishmanicidal activity against promastigote forms and cytotoxicity in J774.A1 macrophages, as well as IC_50_ values and selectivity index (SI).

Compounds	Promastigotes IC_50_ (µM)	% Inhibition at 20 µM	J774 CC_50_ (µM) *	SI (CC_50_/IC_50_) ^a^
**LT-70**	12 ± 0.1	100%	64 ± 5	5.5
**LT-73**	13 ± 0.1	100%	>75 **	>5.8
**LT-75**	10.5 ± 1	100%	>75 **	>7.1
**LT-89**	14 ± 0.1	100%	60 ± 4	4.3
**Amphotericin B**	0.04 ± 0	100%	14 ± 0.3	350

^a^ Selectivity index (SI): ratio between the cytotoxic concentration for 50% of J774 macrophage cells (CC_50_) and the inhibitory concentration for 50% of *Leishmania* promastigotes (IC_50_). “>” indicates no significant inhibitory effect observed up to the highest tested concentration (75 µM). * CC_50_ was previously determined in collaboration with Jacob et al. [20]. 20 µM was the lowest concentration tested in the promastigote dose–response series (20–400 µM). Data represent mean ± SD from two independent biological experiments performed in duplicate. IC_50_ and CC_50_ values were determined by linear regression from concentration–response curves. ** >75 µM indicates the highest concentration tested; true CC_50_ was not reached within this range. Selectivity indices (SI) for axenic amastigotes were estimated using the IC_50_ values obtained for promastigote forms.

**Table 2:** Effect of compounds LT-73 and LT-75 on the mitochondrial membrane potential of *L. infantum* promastigote cells using Rhodamine 123 (Rho 123) staining.

Compounds	Mean	Variation
**Control**	709 ± 29	-
**(1× IC_50_) LT-73**	658 ± 9	−0.07 ± 0.05
**(2× IC_50_) LT-73**	697 ± 7	−0.02 ± 0.03
**(1× IC_50_) LT-75**	1425 ± 106	1 ± 0.23
**(2× IC_50_) LT-75**	1000 ± 21	0.41 ± 0.05
**(1× IC_50_) Amb B**	213 ± 1	−0.70 ± 0.01
**(2× IC_50_) Amb B**	193 ± 3	−0.73 ± 0.02
**Hydrogen Peroxide (H_2_O_2_)**	581 ± 35	−0.18 ± 0.08

## Data Availability

Restrictions apply to the datasets generated in this study. The datasets are not publicly available because they are part of an ongoing research project and additional analyses are still being conducted. Requests for access to the data should be directed to the corresponding author.
